# Is treatment “intensity” associated with healthier lifestyle choices? An application of the dose response function

**DOI:** 10.1016/j.ehb.2016.09.001

**Published:** 2016-12

**Authors:** Eleonora Fichera, Richard Emsley, Matt Sutton

**Affiliations:** a Manchester Centre for Health Economics, University of Manchester, Manchester M13 9PL, United Kingdom; b Centre for Biostatistics, University of Manchester, Manchester M13 9PL, United Kingdom

**Keywords:** Dose-response function, Non-linear dynamic models, Health behaviours

## Abstract

Healthy lifestyle choices and doctor consultations can be substitutes or complements in the health production function. In this paper we consider the relation between the number of doctor consultations and the frequency of patient physical activity. We use a novel application of the Dose-Response Function model proposed by Hirano and Imbens (2004) to deal with treatment endogeneity under the no unmeasured confounding assumption. Our application takes account of unobserved heterogeneity and uses dynamic non-linear models for the treatment and outcome variables of interest. Using seven waves of the British Household Panel Survey, we find that higher treatment intensity and frequency of physical activity are inversely related. We show that accounting for both treatment selection and unobserved heterogeneity halves the size of this relationship. An additional doctor consultation is associated with a 0.5 percentage point reduction in the probability of undertaking vigorous physical activity. Our results hold for a sub-sample visiting the doctor for health check-ups, and are shown to be robust using instrumental variables.

## Highlights

• Physical activity is negatively associated with the lagged number of doctor visits.
• Using a Generalised Propensity Score halves this association.
• This association is robust to using Fixed Effects and instrumental variable models.

## 1 Introduction

Within the World Health Organisation (WHO) European Region, almost 77 percent of the disease burden is due to five major non-communicable diseases (NCD): diabetes, cardiovascular diseases, cancer, chronic respiratory diseases and mental disorders. Amongst its nine global targets to combat these diseases, the WHO has included a reduction of physical inactivity and tobacco consumption, and an increase in treatment and prevention of NCD by primary care doctors (World Health Organization, 2014). There is a wide range of activities that primary care doctors can undertake in treating and preventing NCD, including testing, prescribing and providing lifestyle advice to their patients.

A large literature has investigated the determinants of lifestyle behaviours and contacts with primary care doctors (see for example, Manning et al., 1991, Kenkel, 2000, Chaloupka and Warner, 2000, Fernandez-Olano et al., 2006, Morris et al., 2005). Both forms of health investments have common determinants, including socio-economic and demographic factors, preferences, social networks and information. However, little is known about the interaction between these investments. Our aim is to bring together the literature on the determinants of lifestyle behaviours and healthcare utilisation by examining the association between contacts with primary care doctors and healthy lifestyle choices.

There is a substantial literature showing that health status is positively affected by the supply of doctors (see for example, Aakvik and Holmảs, 2006, Auster et al., 1969, Gravelle et al., 2008, Or et al., 2005, Robst, 2001, Robst and Graham, 1997). Evidence from the U.S., U.K., Norway and a cross-section of OECD countries shows that increasing the number of doctors per capita decreases mortality rates and improves health-related quality of life.

In a Becker-type economic framework, the effect of contacts with doctors on healthy lifestyle choices is ambiguous (Becker, 2007). Individuals invest in their health to equate marginal utility of this investment with its marginal cost. However, there is a trade-off between current costs of healthy lifestyle behaviours (e.g. diverting time and resources away from other activities) and future increased life expectancy. In an application of this model Kaestner et al. (2014) identified two offsetting effects that are applicable to the present study.

On the one hand, there is a “competing risk of death effect” as more contacts with doctors might increase the quantity and productivity of health investments which in turn increase life expectancy and the benefit of investments in health. This leads to a *positive* association between contacts with doctors and healthy lifestyle choices.

On the other hand, Kaestner et al. (2014) pointed out that a “technological substitution effect” might occur if healthy lifestyle choices and contacts with doctors are substitutes in the health production function. This leads to a *negative* association between contacts with doctors and healthy lifestyle choices because more doctor contacts lower the marginal benefit of other health investments.

Although the direction of this association could have important implications for policies that aim to increase access to health care professionals, only one paper has explicitly investigated this empirical question. Schneider and Ulrich (2008) used two waves of the German Socio-Economic Panel Study (GSOEP) to examine the relation between a patient’s health-related behaviour and the probability of visiting a doctor. Patients’ health-related behaviours were measured by an indicator that took a value of one if the respondent was smoking and overweight. They used a recursive bivariate probit model with the exclusion restriction that stress directly affects patients’ health-related behaviour and does not directly affect visits to the doctor. As patients who are overweight and smoke were more likely to visit the doctor, they found evidence of substitutability between visits to the doctor and healthy lifestyle choices.

Doctors can affect patients’ health behaviours by providing lifestyle advice and treatment. Whilst we would expect healthy lifestyle behaviours and lifestyle advice to be either complements or independent of each other, treatment and health behaviours could be substitutes, complements or independent of each other. The only three papers investigating this relationship focused on different target populations and treatment regimens, and found mixed results. Kaestner et al. (2014) used the Framingham Heart Study spanning between 1983 and 2001 to examine the relationship between the introduction and widespread diffusion of statins and health behaviours. They found evidence that statin use is a substitute for healthy diet with a particularly large increase in female obesity (33% of the mean). They also found evidence of an increase in moderate alcohol drinking of about 15% of the mean and a decrease in sedentary activity among men. Using pooled cross-sectional data from the Health Survey for England, Fichera and Sutton (2011) found that prescription of lipid-lowering drugs complemented quitting smoking behaviour in patients with cardiovascular diseases, but smoking cessation advice was not effective in reducing smoking. Fichera et al. (2014) used a unique linkage between three waves of the English Longitudinal Study of Ageing and practice-level data on the volume of treatments delivered by doctors. They decomposed doctors’ effort into an element induced by the payment system and a discretionary element, using an exogenous change in doctors’ remuneration that led them to increase rates of prescription and disease control. They found that increases in the rates of disease control decreased patients’ cigarette consumption.

In this paper we examine the association between the “intensity” of treatment and the level of effort that individuals exert in protecting their own health. We measure treatment intensity as the number of contacts with a primary care doctor and individuals’ health behaviours as the frequency of their physical activity, their smoking and alcohol consumption in seven waves of the British Household Panel Survey. This is a new empirical application of the relation between treatment and healthy lifestyle choices as Kaestner et al. (2014), Fichera and Sutton (2011) and Schneider and Ulrich (2008) did not examine the intensity effect of treatment and Fichera et al. (2014) could only focus on practice-level treatment rates.

Selection into the treatment might confound the relation between intensity of treatment and frequency of physical activity. We attempt to mitigate this problem with a novel application of the dose-response function developed by Hirano and Imbens (2004). Our methodological contribution is to develop a dose-response function in a dynamic panel data model as follows. Firstly, we use a panel grouped count data model of visits to the doctor. Secondly, from this model we obtain the Generalised Propensity Score (GPS) to identify individuals who are predicted to have the same level of treatment but have different actual treatment levels. Finally, we estimate a dynamic random effects (RE) ordered probit outcome model of the frequency of physical activity measured at time (τ+1) including the GPS from the treatment model and frequency of physical activity, both measured at time τ.

This is the first methodological application combining the continuous treatment approach with dynamic panel data models. Identification is provided by comparing individuals with different numbers of contacts with the doctor, but the same predicted “intensity” of contacts based on their personal characteristics. The dose-response function uses the GPS to capture the confounders that affect both visits to the doctor and healthy lifestyle choices. It controls for confounding by (complex functions of) observable factors but does not deal with unobserved confounding. We test the robustness of the results to this limitation using fixed effects models and instrumental variables.

The rest of the paper is structured as follows. Section 2 describes the data and the summary statistics. Details of our econometric methodology are examined in Section 3. Section 4 discusses the results. Section 5 concludes.

## 2 Data and summary statistics

### 2.1 The British Household Panel Survey (BHPS)

The BHPS is an annual survey of each adult (16 years of age and older) member of a nationally representative sample of more than 5000 households, making a total of approximately 10,000 individual interviews.

In this survey individuals are asked “*Since [last 12 months], approximately how many times have you talked to, or visited a GP or family doctor about your own health? Please do not include any visits to a hospital*” with the possible answers being: none; one or two times; three to five times; six to ten times; and more than ten times. Individuals are not asked for reasons for their GP visits.

In the main analysis, we consider physical activity as the proxy for individuals’ investments in their health. All individuals in the survey are asked about the frequency of their physical activity in one of a succession of questions that ask about things people do in their leisure time. As this question is asked every other year from 1996 to 2008, we select seven of the 18 waves of the BHPS. From the question: “*Please […] tell me how frequently you: Play sport or go walking or swimming?*” individuals can choose any of the following: “*At least once a week; At least once a month; Several times a year; Once a year or less; Never/almost never*”. We define physical activity in increasing level of frequency, or effort.

We also consider, as supplementary analysis, smoking and alcohol consumption. Smoking is measured as the average number of cigarettes per day and alcohol drinking is a four scale variable (from drinking at least once a week (1) to once a year or less (4)).

We consider a number of questions on individuals’ ethnic and educational background, gender, age, family composition and marital status, employment, income and wealth, and geographic location. The treatment, medical consultations, and the outcome, frequency of physical activity, are measured at time (τ+1) with respect to these covariates. All the socioeconomic characteristics are considered potential predictors of the treatment and enter the outcome regression either directly or via the GPS.

We consider a number of dummies indicating whether the respondent is white, black, Asian (Indian, Pakistani, Bangladeshi or Chinese) or other ethnic background. A set of dummy variables is included to indicate whether the respondent has obtained a university degree, a high school diploma (the U.K. A-level or O-level), the Higher National Diploma, a semi-professional qualification in the U.K or no qualification at all. We consider the number of children under the age of two years in the household. We dichotomise employment status to indicate whether the respondent is employed (either be employee or self-employed) as opposed to retired, unemployed, on maternity leave or on other employment status. We have taken the natural logarithm of the equivalised value of household monthly income and deflated it by the consumer price index with 1995 as base year. We also considered the number of rooms in the house as an indicator of wealth. A set of dummy variables is included indicating the geographical region of the UK in which the respondent lives.

Respondents are asked to identify the physical health problems and disabilities they are currently suffering from a list of 15 physical health conditions. We group these conditions in an homogenous set of eight dummies: musculoskeletal (e.g. arms, legs, feet and back problems); cardiovascular diseases (e.g. heart problems and high blood pressure); diabetes; skin, head or sight problems; respiratory problems; stomach problems; depression and other conditions. In addition to the type of conditions, we construct a series of five dummies indicating the number of health conditions between 0 and two, three, four, five and over six. In order to mitigate reverse causality both the number and type of health conditions are measured at the first wave in which individuals are interviewed.

In supplementary analyses we focus on a subsample of individuals who have visited the doctor at least once for preventive purposes. This is intended to alleviate concerns that unobserved health conditions affect both the propensity to engage in physical activity and doctor visits. Unfortunately, there is no longitudinal data in the UK that asks patients for the reasons why they have visited the doctor. The English Longitudinal Study of Ageing, like the BHPS, reports the type of conditions diagnosed by the doctor and whether the respondent has visited the doctor, but does not contain the number of doctor visits or the reason for visiting the doctor. The Health Survey for England, a cross-sectional survey held since 1991, asks for the number of doctor visits but not the reason for visiting the doctor. Therefore, we restrict one of our supplementary analyses to BHPS respondents who have undergone at least one health check in the last year.

In a series of questions about which preventive health check-ups respondents have undertaken, we select the National Health Service (NHS) check-ups that are most likely done in a primary care practice. These are blood pressure measurement, cholesterol measurement, cervical screening, breast screening and blood tests. In the supplementary analysis, we restrict the sample to individuals who reported having had at least one of these check-ups in the last year. Not all of the visits in this sample would have been for preventive purposes, but this supplementary analysis is focused on a sub-set of the full sample for which a greater proportion of their visits were for preventive purposes. Nearly 2000 individuals per year (about 94% of the sample) reported having one of these tests, leading to a combined sample of 11,736 observations.

For our instrumental variables analyses we generate two instruments: i) the number of times that the individual’s spouse has visited to a doctor; and ii) the average number of consultations with the doctor in the individual’s Local Authority District (LAD) of residence.

### 2.2 Summary statistics

In the main analysis we consider the population aged between 30 and 59 years because their need for medical consultations and their health effort is expected to differ substantially from the older population. Summary statistics are reported in Table 1.

More than a quarter of this population group have not been to the doctor in the past year. Approximately 37% of respondents visited the doctor once or twice a year and 19% went to the doctor between three and five times a year. More frequent visits to the doctor are rarer, with almost 9% of people going to the doctor six to ten times a year and about 8% of people going more than ten times a year.

Whilst 48% of the sample reported playing sport, walking or swimming at least once a week, 26% of people reported that they never or almost never undertook these forms of physical activity. About 12% of people do these forms of physical activity at least once a month and 9% several times a year.

On average this sample has an equivalised household monthly income of £679 and lives in a house containing five rooms. About 78% of the population is either an employee or self-employed. Approximately 16% of people report having at least a university degree and 51% report to have obtained a high school diploma.

The population aged between 30 and 59 years is relatively healthy, with 93% having at most two health conditions and only 2% of people reporting having six conditions or more. About 16% of people report having a type of musculoskeletal problem and 17% report skin, head or sight problems.

## 3 Econometric methodology

Our empirical strategy has two main features. Firstly, we predict the propensity score from a (panel) grouped count data model to account for selection of the intensity of treatment, as the number of visits to the doctor depends on individuals’ previous behaviour and socioeconomic characteristics. Secondly, we also account for non-linearities and persistency in the effort that individuals exert on their health investments with panel data ordered probit models.

Matching methods have been widely used in the programme evaluation literature of the last two decades (see Augurkzy and Kluve, 2007 for an overview). This is largely due to their ability to mimic experimental settings *ex post*. As many observational studies involve non-binary treatments, recent literature has extended propensity score methods to the cases of multi-valued treatments (Imbens, 2000, Lechner, 2001), and, more recently, continuous treatments (Behrman et al., 2004, Hirano and Imbens, 2004, Imai and van Dyk, 2004). Hirano and Imbens (2004) apply a generalisation of the binary treatment propensity score, namely the *generalised propensity score* (GPS), to a population of individuals winning the Megabucks lottery in Massachusetts in the mid-1980. They estimate a *dose-response function* (DRF) for the amount of lottery prize wins on subsequent labour earnings using the propensity score to adjust for differences in pre-treatment characteristics.

In this section, we build on the approach developed by Hirano and Imbens (2004). As in Hirano and Imbens (2004) application, the “intensity” of treatment depends on pre-treatment characteristics. We therefore compare individuals with similar pre-treatment characteristics and similar GPS, i.e. *predicted* levels of treatment, but different *actual* treatment levels.

We contribute to the original study by Hirano and Imbens (2004) by developing a dose-response function in a dynamic panel data model as follows. Firstly, we use a (panel) grouped count data regression to model visits to the doctor. Secondly, we obtain the GPS from a panel model conditional on a variety of socioeconomic characteristics. Finally, we estimate a panel random effects (RE) ordered probit model of the outcome measured at time (τ+1) including the GPS from the treatment model and the value of the outcome measured at time τ.

### 3.1 Implementation and estimation

Let us define *N* as a random sample for the population of private households in the UK. For each individual *i* in this sample we observe a set of pre-treatment covariates (i.e. measured at time τ before the treatment). These are the $X_{i\tau }$ or the $X_{i0}$ variables described in Section 2.

A number of econometric methodologies have been used to model utilisation of primary care with the majority of these studies adopting binary and count data models (see for example, Cameron and Trivedi, 1986, Cameron and Trivedi, 1993, Cameron and Windmeijer, 1996, Jones, 2000, Sarma and Simpson, 2005). Count data models allow the distribution of doctors’ visits to be skewed and restrict its predicted values to be non-negative. As we only observe intervals of visits, we base our estimation on a generalisation of count data models to grouped data (see for example, Moffatt and Peters, 2000, Teckle and Sutton, 2008, Brown et al., 2014). In order to account for grouping we modify the log-likelihood function as follows:

(1) $$log\text{L}\left(\beta \right)=\overset{n}{\underset{i=1}{\sum }}\overset{J}{\underset{j=1}{\sum }}\overset{\Xi }{\underset{\tau =1}{\sum }}d_{ij\left(\tau +1\right)}\text{log}\left[P\left(T_{i\left(\tau +1\right)}\in I_{j}\right)\right]$$

where $T_{i\left(\tau +1\right)}$ indicates the number of visits to the doctor which is forward looking with respect to the covariates and $I_{1},\ldots ,I_{J}$ indicates each of the five groups of visits to the doctor with each group containing a set of consecutive integers $\left\{a_{j},a_{j+1},\ldots a_{j+k}\right\}$; the indicator $d_{ij\left(\tau +1\right)}$ takes the value one if $t_{i\left(\tau +1\right)}\in I_{j}$ and zero otherwise; and $P\left(T_{i\left(\tau +1\right)}\in I_{j}\right)$ is the probability that individual *i* reports group *j* modelled with a Poisson distribution^1^:

(2) $$P\left(T_{i\left(\tau +1\right)}=t_{i\left(\tau +1\right)}\right)=\frac{\text{exp}\left(-\lambda_{i\left(\tau +1\right)}\right)\lambda_{i\left(\tau +1\right)}^{t_{i\left(\tau +1\right)}}}{t_{i\left(\tau +1\right)}!}$$

with $\lambda_{i\left(\tau +1\right)}=\text{exp}\left(\beta X_{i\tau }+\gamma \left[T_{i\tau }=t_{i\tau }\right]+c_{i}\right)$ where we assume the distribution of $c_{i}$ to be normal. We follow Wooldridge (2005) and parameterise the time-invariant component as $c_{i}=\delta 1.\left[T_{i0}=t\right]+\vartheta {\bar{X}}_{i}+\eta_{i}$ where $T_{i0}=t$ indicates the initial condition of each realised value of the visits to the doctor and $\eta_{i}$ is the new unobserved time-invariant effect assumed to be normally distributed. The Chamberlain (1984) and Wooldridge (2005) approach allows us to account for potential endogeneity of the initial conditions by including individuals’ background characteristics over the entire observed period.^2^ ${\bar{X}}_{i}$ is the average of the time-varying pre-treatment characteristics described in Section 2. As suggested by Cameron and Trivedi (2015) this can be simply estimated by entering ${\bar{X}}_{i}$ as additional regressor.

The choice of covariates depends on the behavioural factors that affect healthcare utilisation. Education, a proxy for human capital, is related to both health knowledge and self-management (Goldman and Smith, 2002, Cutler and Lleras-Muney, 2006). Income and employment status are proxies for the opportunity cost of time of visiting the doctor. As doctor consultations do not attract user charges in the UK, this is the only cost to the individual. There is also some evidence that ethnicity affects the utilisation of primary care in England (Goddard, 2008). Finally, we consider the number and type of health conditions measured in each individual’s first year of observation. As there might be interactions between these characteristics, we include interactions between types of conditions, age and education. This categorisation of these pre-treatment variables achieves good balance between the treatment and comparison groups.

Each individual *i*’s prediction from this regression is constrained to lie within the observed group $I_{j}$ to obtain the “predicted” medical consultations $T_{i\left(\tau +1\right)}={\hat{\lambda }}_{i\left(\tau +1\right)}$. In other words, let the upper and lower bound of each interval group $I_{j}$ be u and l, respectively. The “predicted” medical consultations $T_{i\left(\tau +1\right)}$ is calculated as*:*

$$\begin{array}{ccc} T_{i\left(\tau +1\right)}=l & if & \beta X_{i\tau }+\gamma \left[T_{i\tau }=t_{i\tau }\right]+\varepsilon_{i\left(\tau +1\right)}\leq l \end{array};$$

$$\begin{array}{cccc} T_{i\left(\tau +1\right)}=u & if & \beta X_{i\tau }+\gamma \left[T_{i\tau }=t_{i\tau }\right]+\varepsilon_{i\left(\tau +1\right)}\geq u & and \end{array}$$

$$\begin{array}{ccc} T_{i\left(\tau +1\right)}=\beta X_{i\tau }+\gamma \left[T_{i\tau }=t_{i\tau }\right]+\varepsilon_{i\left(\tau +1\right)} & if & l<\beta X_{i\tau }+\gamma \left[T_{i\tau }=t_{i\tau }\right]+\varepsilon_{i\left(\tau +1\right)}<u \end{array}$$

with $\varepsilon_{i\left(\tau +1\right)}$ including the time invariant component $c_{i}$.

We then define the value of the outcome variable, the frequency of physical activity, associated with these medical consultations as $Y_{i\left(\tau +1\right)}$. For each individual *i* there exists a set of potential outcomes at each time $\left(\tau +1\right),{\left\{Y_{i\left(\tau +1\right)}\left(t\right)\right\}}_{t\in \tau }$ defined by Hirano and Imbens (2004) as the unit-level dose-response function. Whilst in the binary case τ={0,1}, in the continuous case τ is an interval $\left[t_{0},t_{1}\right]$. We are interested in the average dose-response function (ADRF), $\mu \left(t\right)=E\left[Y_{i\left(\tau +1\right)}\left(t\right)\right]$.

The propensity function is defined by Hirano and Imbens (2004) as the conditional density of the actual treatment given the observed covariates, that is, $r\left(t,x\right)=f_{T|X}(t|x)$. The GPS is then defined as R=r(T,X).

The GPS has a balancing property similar to that of the binary treatment propensity score, that is, within strata with the same value of r(t,x), the probability that T=t does not depend on the value of X. More formally, let D(t)=1.[T=t], then X⊥D(t)|r(t,x). Hirano and Imbens (2004) show that, in combination with a suitable unconfoundedness assumption, this balancing property implies that assignment to treatment is also unconfounded, given the GPS. In this case, a generalisation of the unconfoundedness assumption for binary treatment made by Rosenbaum and Rubin (1983) states that assignment to treatment is weakly uncounfounded for a given set of pre-treatment covariates, that is, Y(t)⊥D(t)|Xforallt∈τ. The weak unconfoundedness hypothesis is based on pairwise independence of the treatment with each potential outcome. It also requires Y(t) and the treatment to be “locally” independent at the treatment level of interest T=t, not T (Imbens, 2000). This is a key point of our identification strategy as it assumes no unobserved confounding between our outcome and treatment of interest.

The estimation of the dose-response function (DRF) consists of three stages, which will now be explained in turn.

#### 3.1.1 First stage – treatment model and the balancing test

In the first stage, we estimate the time-varying score $R_{i\left(\tau +1\right)}=r\left(T,X\right)$. We modify the linear model used by Bia and Mattei (2008) and estimate the score from the same dynamic correlated random effects grouped Poisson model in (1), but the predicted score is not constrained to be within the bounds of the group in which the individual originally reported. Whereas $T_{i\left(\tau +1\right)}$ represents the best prediction conditional on pre-treatment covariates of the actual visits to the doctor within each reported group, the conditional expectation of the treatment used to retrieve the GPS represents the visits to the doctor an individual would be expected to make given her pre-treatment characteristics, which may lie outside the bounds of the group in which the actual treatment level lies.

From the grouped count data model we estimate the GPS, ${\hat{R}}_{i\left(\tau +1\right),}$ from the Poisson distribution defined in (2). We follow Hirano and Imbens (2004) and balance the covariates *blocking* on both the treatment variable, the number of visits to the doctor $\left(T_{i\left(\tau +1\right)}\right)$, and on the estimated GPS. We implement this procedure by first dividing the sample into three cuts according to the distribution of the actual treatment, namely, those who never visit the doctor, those who visit the doctor between one and five times, and six times or more a year. As suggested by Hirano and Imbens (2004) we chose the cut-off points to fit the distribution of visits.

Within each cut, we compute the GPS from Eq. (2) at the median of each cut of the treatment. Then, we divide each cut into blocks defined by tertiles of the GPS evaluated at the median, considering only the GPS distribution of individuals in that particular cut of medical consultations. Within each block we calculate the mean difference of each covariate between individuals who belong to a block of the cut and those who belong to other cuts. We combine all the mean differences by using a weighted average with weights given by the number of observations in each tertile of the GPS. This procedure is repeated for each of the cuts and for each pre-treatment characteristic. The key assumption of the first stage is that, *conditional* on the GPS, there are no statistically significant differences between the characteristics of individuals belonging to different treatment intervals. This does not necessarily imply that there are no differences in their unobserved characteristics.

#### 3.1.2 Second stage – the outcome model

The second stage is to model the conditional expectation of the frequency of physical activity $Y_{i\left(\tau +1\right)}$, given the visits to the doctor $T_{i\tau }$, and the GPS,$R_{i\tau }$, as follows:

(3) $$Y_{i\left(\tau +1\right)}^{*}=\alpha_{0}+\alpha_{1}T_{i\tau }+\alpha_{2}R_{i\tau }+\alpha_{3}R_{i\tau }^{2}+\gamma_{1}1.\left[Y_{i\tau }=m\right]+\gamma_{2}1.\left[Y_{i0}=m\right]+\eta_{i}+\varepsilon_{i\left(\tau +1\right)}$$

where we include a second-order polynomial of the GPS. Eq. (2) is estimated as a dynamic random effects ordered probit model with $Y_{i\left(\tau +1\right)}^{*}$ being the latent propensity to put effort on physical activity, $\eta_{i}$is the individual-specific, time-invariant random component capturing the unobserved heterogeneity of individual *i* and $\varepsilon_{i\left(\tau +1\right)}$ is the time-variant error term. We use a dynamic random effects ordered probit model to alleviate concerns about unobserved confounding. However, we note that this involves assumptions of strict exogeneity and orthogonality between $\eta_{i}$ and the regressors.

The observed measure of frequency of physical activity is related to $Y_{i\left(\tau +1\right)}^{*}$ as follows:

(4) $$Y_{i\left(\tau +1\right)}=m\text{if}\delta_{m-1}<Y_{i\left(\tau +1\right)}^{*}<\delta_{m}\text{for}\text{m}=1,\ldots ,\text{M}$$

where individual *i* reports the *m*th frequency of physical activity, with *M* being the healthiest option (i.e. *M* *=* *5* indicates playing sport, walking or swimming at least once per week). If the underlying latent propensity $Y_{i\left(\tau +1\right)}^{*}$ is between $\delta_{m-1}$and $\delta_{m}$, the realised value of frequency of physical activity is $m=\left(Y_{i\left(\tau +1\right)}=m\right).$

The term $Y_{i\tau }=m$ and $Y_{i0}=m$ indicate, respectively, the frequency of physical activity prior to $Y_{i\left(\tau +1\right)}$ and the initial condition of each realised value of the frequency of physical activity.

We calculate the average partial effects of the visits to the doctor $T_{i\tau }$ for each realised outcome as $APE\left(T_{i\tau }\right)=\frac{\partial \text{Pr}(Y_{i\left(\tau +1\right)}=m|T_{i\tau },R_{i\tau })}{\partial T_{i\tau }}$.

#### 3.1.3 Third stage – the DRF plot

The last stage consists of estimating the DRF at each level of the treatment as follows:

$$E\left[{\hat{Y}}_{i\left(\tau +1\right)}\left(t\right)\right]=\frac{1}{N}\overset{N}{\underset{i=1}{\sum }}\hat{\beta }\left(t,\hat{r}\left(t,X\right)\right)$$

where $\beta \left(t,r\right)=E\left[Y_{i\left(\tau +1\right)}|T_{i\tau }=t_{i\tau },R_{i\tau }=r_{i\tau }\right]$.

This procedure averages over the score evaluated at the treatment level of interest r(t,X). Hirano and Imbens (2004) point out that a causal interpretation can be given to the value of the DRF for treatment value *t*, $E\left[{\hat{Y}}_{i\left(\tau +1\right)}\left(t\right)\right]$, compared to the other treatment level *t*’. This is based on the maintained assumption that there is no unobserved confounding between the outcome and treatment of interest.

### 3.2 Supplementary analyses

We also undertake a range of supplementary analyses. Some of these check the robustness of the results to the model specification; others focus on an alternative sample of individuals who have visited the doctor at least once for preventive purposes and implement alternative econometric techniques.

#### 3.2.1 Alternative model specifications

We modify the main model specification in four ways. First, we modify the RE ordered probit model to include all the covariates used to estimate the GPS directly in the outcome model. Second, we modify the definition of treatment in the outcome models to include the number of doctor visits: i) treated as count variable; ii) with a set of dummies for each interval of visits; and iii) the prediction from the interval regression rounded to the closest integer. Third, we adopt the stratification method suggested by Imai and van Dyk (2004) by estimating the outcome model separately for each tertile of the GPS and then take the weighted average of the coefficients. Finally, we repeat the analysis described in Section 3.1 including the past frequency of physical activity in the treatment model. In this analysis we lose one year of data for the outcome model.

Other specifications provide wider evidence to support the plausibility of the findings. We repeat the analysis for two other health behaviours, smoking and alcohol drinking. We consider the same years used for estimating the physical activity outcome models (i.e. every other year between 1996 and 2008) and focus only on those participating at some level in these behaviours in the first year of observation. We estimate treatment and outcome models for the older population aged 60 and over.

#### 3.2.2 Refinement of treatment

There is a concern that reverse causation might still bias our results even after controlling for past physical activity, carefully selecting the timing between outcome and treatment, and analysing other health behaviours. We attempt to address this concern by selecting a sub-sample of individuals who we know have visited a doctor for preventive activity by undertaking at least one check-up. There is no other micro-level longitudinal data in the UK that asks patients the reason for visiting the doctor. So whilst this is only an attempt to check the robustness of our estimates for a sub-sample of BHPS respondents who access preventative health services, we acknowledge that they might have visited the doctor for other reasons as well. We have not found any recent aggregate figures for the whole of the UK reporting summary statistics on the reasons why people visit the doctor. However, we found that a 2013 study by the Information Service Division in Scotland reported that amongst the ten activities that attract most of the consultations in primary care practices are blood testing (500 consultations per 1000 population), blood pressure monitoring (350 consultations per 1000 population) and general diagnostic tests (210 consultations per 1000 population). Prescription or medication review account for about 60 consultations per 1000 population. This might suggest that most consultations are for preventive or monitoring purposes. Using Kaestner et al. (2014) theoretical model, for this group of people we can think of visits to the doctor and healthy lifestyle choices as two preventive activities in their health production function. A “technological substitution effect” might prevail if doctor contacts lower the marginal benefit of other health investments. For the sub-sample of people who have at least one check-up we re-estimate a RE ordered probit model including all the covariates in the treatment model.

#### 3.2.3 Alternative econometrics techniques

An additional major concern is that dose response models cannot deal with unobserved confounding. The balancing test conditional on the GPS shows that individuals are similar in a wide range of observable characteristics, but it is still possible that they differ in some unobserved components that we have not controlled for. For instance, individuals might differ in their propensity to visit the doctor and this propensity might affect their propensity to engage in physical activity. Additionally, the RE models assume that the unobserved component $\eta_{i}$ is uncorrelated with the regressors.

We address this concern in two ways. First, we estimate a linear fixed effects model under the assumption that unobserved heterogeneity is time invariant. Fixed effects models have the advantage to relax the RE assumption of orthogonality between $\eta_{i}$ and regressors. Secondly, we use two separate instruments in a two-stage residual inclusion (2SRI) model, an alternative implementation of the two-stage least squares model that is consistent in non-linear models (see for example, Terza et al., 2008). 2SRI models have been recommended for the estimation of count data models (see Wooldridge, 1997, Wooldridge, 2002).

The first stage is identical to Eq. (1) with the exception of the inclusion of two instruments in two separate models: i) the number of doctor consultations by the individual’s spouse, denoted by $T_{-i\tau }$; or ii) the average number of consultations in the individual’s Local Authority District (LAD) of residence. These are relevant instruments because they are correlated with doctor visits in the first stage model. However, we note that both instruments have advantages and disadvantages. On the one hand, the estimation sample when using spousal consultations with the doctor is restricted to married or cohabiting respondents for whom we have information on their spouses (about 67% of our main sample of individuals). On the other hand, there is more variation in using individual *i*’s spouse than her location. There is however a fair amount of variation in the LAD-measure of doctor consultation with a standard deviation of about one (on the 0–4 scale). Finally, whilst one might be concerned that spousal visits to the doctor might affect individual *i*’s engagement in physical activity for the sharing of time resources (e.g. if *i* had to take his/her spouse to the doctor), it is unlikely that the LAD *average* of doctor consultations directly affects her own engagement in physical activity. It should be a valid instrument and alleviate concerns of reverse causality and omitted variable bias because the average LAD medical consultations should be uncorrelated with individual *i*’s unobserved determinants of physical activity.

The second-stage is a random effects ordered probit model of physical activity:

(5) $$Y_{i\left(\tau +1\right)}^{*}=\alpha_{0}+\alpha_{1}T_{i\tau }+\beta X_{i\tau }+\gamma {\hat{u}}_{i\left(\tau +1\right)}+\varepsilon_{i\left(\tau +1\right)}$$

where $Y_{i\left(\tau +1\right)}^{*}$ indicates the propensity to engage in some level of physical activity with realised value as defined in Eq. (4). ${\hat{u}}_{i\left(\tau +1\right)}$ is the error term predicted in the first stage. We have bootstrapped the standard errors with 1000 replications. When the average LAD medical consultations are used as instrument, we cluster standard errors by the LAD.^3^ For comparability with our previous estimates, we have estimated Eq. (5) on the same sample used for Eq. (3). $T_{i\tau }$ indicates the number of doctor visits as defined in the previous models. A large difference between the coefficients of doctor visits, $T_{i\tau }$, instrumented by either measure could indicate whether any of the above mentioned caveats are a serious concern for our identification strategy.

## 4 Results

### 4.1 Main analyses

**Fig. 1:**
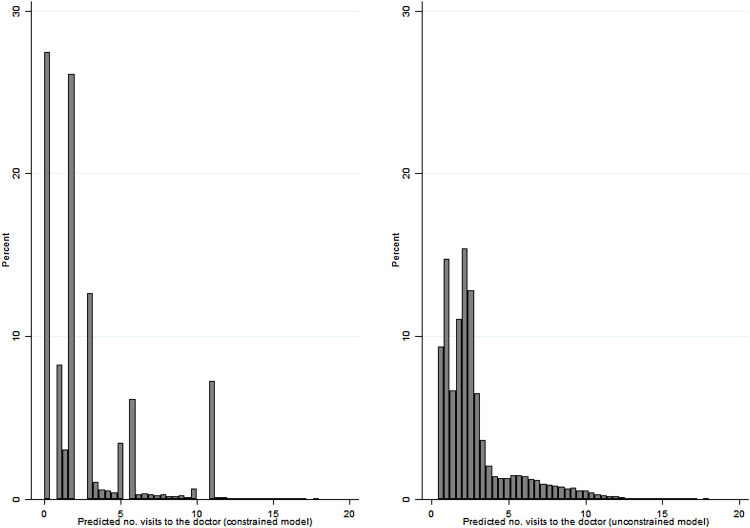
Predicted visits to the doctor. Note: Variable in the left panel has been predicted from constrained grouped Poisson model; variable in the right panel has been predicted from unconstrained grouped Poisson.

On the left panel of Fig. 1 we display the histogram of $T_{i\tau }$, the number of visits to the doctor predicted from the constrained correlated random effects dynamic grouped Poisson model in Eq. (1).^4^ The spikes indicate the lowest bounds of the original treatment group to which we attribute predicted values that lie outside the reported group. On the right panel of Fig. 1 we display the histogram of the predicted visits to the doctor from the unconstrained treatment model described in Subsection 3.1.1. The difference between the two distributions indicates there are individuals who reported a level of treatment intensity within a given interval but would not have been predicted to do so based on their demographic, initial health and socio-economic characteristics.

We report the results of each of the three stages for estimating the DRF, namely, the treatment model and the balancing test, the outcome model and the DRF plot.

The results of the treatment model are reported in Table 2. We briefly comment on it as it is only used to estimate the GPS. We find that people with higher income are more likely to visit the doctor indicating the income effect prevails over the substitution effect. Visits to the doctor measured at time (τ+1) are an increasing function of visits measured at time τ indicating a strong state dependence. Most initial health conditions have a positive association with visits to the doctor except CVD. As the interaction term of CVD with university degree^5^ is positive and statistically significant, the coefficient of the initial CVD condition refers to those without a university degree. A recent paper by Labeit et al. (2013), using the same data as the present study and dynamic panel data probit models, found that people with lower education are less likely to uptake cholesterol tests and blood pressure checks. This might confirm our result that there are educational differences in healthcare utilisation particularly for those with cardiovascular conditions.

Table 3, Table 4 report the balancing tests for each of the three cuts of the treatment, unadjusted and adjusted for the GPS, respectively. We report a more conservative significance value at the one percent level because of multiple comparisons over 70 variables. Table 3 shows a high level of statistical imbalance in most of the pre-treatment covariates for each of the cuts. Imbalance is especially high when considering socio-economic characteristics, and the initial types and number of health conditions and visits. This indicates that BHPS respondents who visit the doctor more or less frequently differ in their observed characteristics. In Table 4 we show that after adjusting for the GPS we obtain a very good balance for all of the pre-treatment characteristics. This indicates that *conditional* on the GPS, BHPS respondents visiting the doctor more or less often are similar to each other. However, we cannot assert that they have similar unobserved characteristics.

Table 5 reports the coefficients of the outcome models. We first compare the random effects dynamic ordered probit model (Model IV) to the pooled and static model (Models I–III) and then we discuss the size of these effects in Table 6. Each model includes visits to the doctor measured at time τ, but whilst model (I) omits the GPS, all the other models include it. Model (IV) additionally includes frequency of physical activity measured at time τ and its initial conditions. Each of these models makes a different assumption about unobserved heterogeneity with the dynamic RE model being more complex by allowing for state dependence. *Ex-ante* we expect the size of the relationship between treatment “intensity” and physical activity to be smaller as we account for unobserved heterogeneity and state dependence.

The statistical significance of the GPS in model (II) indicates that in model (I) there were omitted factors affecting both the higher propensity to visit the doctor and frequency to do physical activity. There is evidence of non-linear effects of the GPS as its higher order terms significantly affect the frequency of physical activity. The size of the GPS is smaller when unobserved heterogeneity is accounted for in Models (III) and reduces by almost half when both unobserved heterogeneity and state dependence is accounted for in the dynamic model (IV). Physical activity measured at time τ is positively associated with frequency of physical activity measured at time (τ+1) as compared to no activity. This association is stronger the more frequently the respondent played sport, walked or swam at time τ, indicating very strong state dependence.

As we cannot directly interpret the magnitude of the coefficients in non-linear models, we report marginal effects in Table 6. We compare the marginal effects from Model (III), the static RE model with GPS, to the marginal effects from Model (IV), the dynamic RE model with GPS. We show that the size of the effect estimated by Model (III) is double that estimated by Model (IV) for frequency of physical activity between once a year and once a week.

The marginal effects from Model (IV) show that treatment is associated with a shift of the distribution of physical activity to the left. An additional medical consultation is associated with a decrease in the probability of engaging with physical activity at least once a week by 0.5 percentage points while the probability of not doing any physical activity at all increases by 0.4 percentage points. The changes in moderate physical activity are smaller as, on average, an additional medical consultation is associated with an increase in the probability to engage in physical activity between once a year and once a month by almost 0.05 percentage points.

**Fig. 2:**
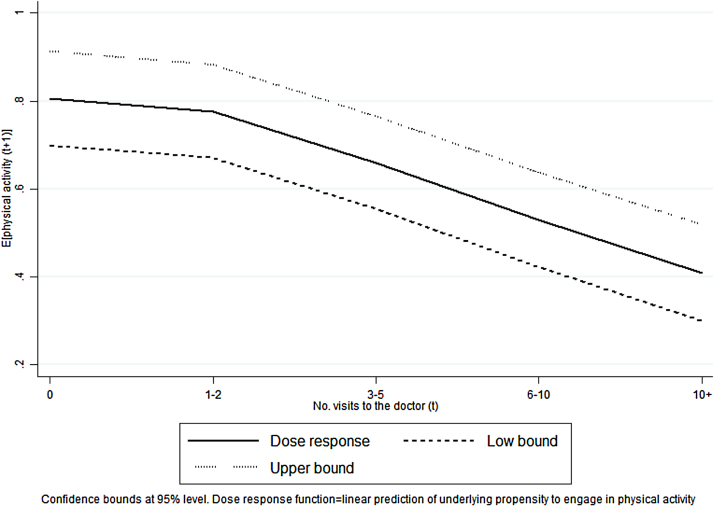
Plot of the dose response function.

Fig. 2 displays the linear predictions of physical activity from the second stage non-linear regression with a range between zero and one. The figure shows a general reduction in the level of physical activity as the intensity of treatment increases. The slope of the DRF is steeper for more than two visits to the doctor. This indicates a negative association between doctor visits and frequency of physical activity that becomes stronger at higher levels of intensity.

### 4.2 Supplementary analyses

In Table A1 we report estimates when including all the covariates from the GPS regression directly in the outcome equation. The association between physical activity and number of visits to the doctor remains negative and statistically significant and the size of the coefficient is quite close to the one in Model (IV) of Table 5. Including the GPS in the outcome model reduces the curse of dimensionality and improves efficiency because we can just use a term (i.e. the GPS) predicted from a treatment model that includes interactions between covariates and polynomial terms instead of individual covariates.

As shown in Fig. 1 doctor visits have been predicted from a constrained model where only the bounds of the intervals map the actual visits. There might be a concern that this prediction is driving our results. In Table A2 we show this is not the case as the same negative association between visits to the doctor and physical activity holds when treatment is defined as a count variable (Model I), as a set of dummy variables (Model II) or as a prediction from the constrained regression rounded to the closest integer (Model III). Model II shows that the association between doctor visits and physical activity is steeper at higher intensity levels as reported in the DRF plot.

Table A3 shows that the relationship between treatment intensity and physical activity is similar when we estimate the outcome model separately by GPS tertiles. In Table A4 we report the results of the outcome model where GPS has been obtained from a treatment model that includes physical activity. We show that our previous results were not driven by the omission of past physical activity from the treatment model as the coefficient of interest has a similar size and statistical significance. This should alleviate concerns about reverse causality.

We find a statistically significant relationship between smoking or drinking and number of visits to the doctor. Table A5 indicates that more frequent visits to the doctor are associated with more drinking and smoking, a similar association to the one found for physical activity.

In Table A6 we report alternative specifications to alleviate concerns of reverse causality and unobserved confounding. All models show that there is still a negative association between doctor visits and physical activity. We report in Table A6 the set of covariates that is shared across all models. Model (I) is a RE ordered probit model estimated on the sample of those who undertake at least one health check. The size of the coefficient is very similar to the one in our preferred specification of a dynamic RE model with the GPS in Table 5. We have also estimated Model (I) using all the specifications in Table 5 and results are very similar (available from the authors on request). Model (II) is estimated with a linear FE model where only time varying covariates have been included. The coefficient on doctor visits is also very similar to our previous specifications.

Models (III–IV) report the second stage RE ordered probit coefficients of the 2SRI specification described in Eq. (5). The negative association between number of visits to the doctor and physical activity holds when using either spousal visits to the doctor (Model III) or area average visits (Model IV) as instruments. The similarity of the coefficients on doctor visits using these alternative instruments goes some way to alleviate concerns over their limitations. The coefficient on the residuals predicted from the first stage regression is statistically significant indicating endogeneity of visits to the doctor (Terza et al., 2008). It can be interpreted as evidence that those who have a higher propensity to go to the doctor have a lower propensity to engage in physical activity. As the first stage estimates of these models are similar to those reported in Table 2, we do not report them here, but they are available on request. Both instruments are relevant instruments as they are positively and statistically significantly (at the one percent level) associated with individual’s *i* visits to the doctor (with a coefficient of 0.02 and 0.14, respectively; and a value of the *z* statistics greater than 10).

In Table A7 we show that the negative association between visits to the doctor and physical activity holds for the older population as well. The magnitude of this relation is higher than the one found for the sample aged between 30 and 59 years.

## 5 Conclusions

Healthy lifestyle choices and medical consultations can be substitute or complements in the health production function. Although previous literature (Kaestner et al., 2014, Schneider and Ulrich, 2008) has found evidence of substitutability, medical treatment was measured as a dichotomous variable in these applications. In this paper we have examined the effect of increasing treatment “intensity”, the number of doctor contacts, on frequency of physical activity using seven waves of the BHPS.

We have found evidence of a negative association between treatment intensity and physical activity. This relationship is stronger the higher the intensity of treatment. An additional medical consultation is associated with a reduction in the probability of engaging in physical activity at least once a week by 0.5 percentage points while the probability of not doing any physical activity at all increases by 0.4 percentage points. This association is related to a shift of the distribution of physical activity to the left towards lower frequency of engagement. The changes in moderate physical activity are smaller as, on average, an additional medical consultation is associated with an increase in the probability to engage in physical activity between once a year and once a month by about 0.05 percentage points.

We have also shown that a simple regression of the number of visits to the doctor on the frequency of physical activity suffers from selection bias and over-estimates the relation between medical consultations and investments in health. We have attempted to mitigate this selection bias problem with a novel application of the dose-response function developed by Hirano and Imbens (2004) that combines the continuous treatment approach with dynamic panel data models.

Our novel methodological application has produced three insights in the modelling of the relation between treatment intensity and healthy lifestyle choices. Firstly, we have shown that selection bias accounts for part of the relation between treatment intensity and healthy lifestyle choices as there is a 14% reduction of the coefficient of treatment intensity when the generalised propensity score (GPS) is included in the regression. Secondly, the dose-response function with the GPS could lead to efficiency gains as it allows confounders to enter flexibly in the outcome model via the GPS that can then be stratified and modelled with higher polynomial orders. Our results suggest that accounting for non-linearities in the characteristics determining treatment selection is important as the second-order polynomial of the GPS is statistically significant in the outcome regression. Finally, combining dynamic models and a dose-response function with the GPS has the advantage to flexibly account for treatment selection, unobserved heterogeneity and the dynamic nature of healthy lifestyle choices. We have found the size of selection bias is lower as there is an almost 42% reduction in the coefficient of treatment intensity when we estimate the dose-response function in a dynamic random effects model (i.e. including both the GPS and the lagged values of the outcome variable).

One limitation of our paper is that the measure of frequency of physical activity is only limited to playing sports, swimming or walking. Whilst this is the only type of physical activity that is consistently measured across the BHPS sample, we have shown evidence of a negative association between treatment intensity and other healthy lifestyle behaviours such as reducing cigarettes and alcohol consumption.

A second limitation which we share with the study by Hirano and Imbens (2004) is the lack of exogenous variation in treatment. The application by Hirano and Imbens (2004) focused on a cross-section of lottery winnings which although exogenous belong to a particular selected sample of players. Although we combine dynamic panel data models with the GPS, we are cautious in asserting we are estimating a causal effect. We have attempted to mitigate this limitation by using 2SRI models with spousal and area-average visits to the doctor as instruments. Both instruments have advantages and disadvantages relating to the amount of variation and the potential for direct pathways to physical activity. Under the assumption of time-invariant unobserved heterogeneity we have estimated a FE model. Although our results are robust to both specifications, we note that time-varying unobserved heterogeneity and omitted variable bias might still be possible.

A third limitation is that, in following Bia and Mattei (2008), Hirano and Imbens (2004) and Imbens (2000), we do not correct the standard errors for the inclusion of the GPS in the outcome model.

A final limitation is that we cannot determine what elements of the treatment generate an inverse relationship with healthy lifestyle choices, as the dataset contains no information on the cause and content of doctor consultations. The non-linear association between doctor visits and frequency of physical activity might be concerning if an unobserved (to us as researchers) health problem has induced patients to initiate a doctor visit. This would generate a non-linear and reverse causal association between doctor visits and frequency of physical activity. There is no longitudinal survey data in the UK that asks respondents the reason for visiting the doctor. Instead, we have shown that our results are robust to restricting our sample to individuals who have had at least one preventative health check-up. Official statistics suggest that the majority of doctor consultations are for preventive purposes. These two pieces of information point to the direction of a substitution between two preventive activities in the health production function. However, we highlight that data do not allow us to ascertain the reason for visiting the doctor and therefore we cannot give a causal interpretation to the estimates produced in this study.

## Figures and Tables

**Table A1:** Coefficients of random effects ordered probit model for physical activity measured at (τ+1) with all covariates included.

	Coeff.	Std. Err.
No. visits to the doctor	−0.019***	0.004
Age 45+	−0.108***	0.04
Male	−0.013	0.07
White	0.754**	0.29
Black	−0.558	0.39
Asian	0.148	0.347
Non-single	0.020	0.05
University degree	0.420***	0.05
High school diploma	0.207***	0.03
No. children 0–2	−0.08**	0.04
Employed	−0.014	0.03
Log(Income)	0.085***	0.02
No. rooms in the house	0.048***	0.01
No. initial visits to the doctor (1–5)	0.047	0.03
No. initial visits to the doctor (6+)	−0.086*	0.05
No. initial health conditions (3)	−0.187**	0.08
No. initial health conditions (4)	−0.269**	0.12
No. initial health conditions (5)	−0.193	0.24
No. initial health conditions (6+)	−1.045***	0.32
Initial musculoskeletal condition	−0.224***	0.05
Initial CVD condition	0.132	0.20
Initial diabetes condition	−0.201	0.18
Initial skin, head and sight problems	−0.007	0.05
Initial respiratory problems	0.004	0.06
Initial stomach problems	−0.133	0.08
Initial depression condition	−0.163***	0.05
South East	0.042	0.05
South West	0.171***	0.06
East Anglia	0.147*	0.09
East Midlands	−0.093	0.06
West Midlands	−0.071	0.07
North West	−0.114*	0.06
Yorkshire	−0.040	0.06
Rest of the North	−0.049	0.07
Wales	0.007	0.05
Scotland	0.174***	0.05
2000	−0.043	0.03
2002	0.094***	0.03
2004	0.073***	0.03
2006	0.102***	0.03
2008	−2.421***	0.04

No. observations	27,715	
No. individuals	8489	

*Note*: The BHPS sample consists of head of household + Partner/Spouse every other year 1996–2008. Outcome variable is forward-looking and all covariates measured at time τ. Number and type of conditions are measured at initial time τ. Interactions between health conditions, age and education not displayed.

***p < 0.01; **p < 0.05; *p < 0.1.

**Table A2:** Coefficients of random effects ordered probit model for physical activity measured at (τ+1) with different definitions of treatment.

	Model (I)	Model (II)	Model (III)
No. visits to the doctor (continuous)	−0.049*** (0.007)	–	–
No. visits to the doctor (0)	–	0.058*** (0.02)	–
No. visits to the doctor (1–2)	–	−0.012 (0.02)	–
No. visits to the doctor (3–5)	–	−0.067*** (0.03)	–
No. visits to the doctor (6–10)	–	−0.245*** (0.03)	–
No. visits to the doctor (rounded)	–	–	−0.018*** (0.003)
GPS	0.921*** (0.19)	0.810*** (0.19)	0.787*** (0.20)
GPS squared	−1.493*** (0.36)	−1.190*** (0.36)	−1.257*** (0.37)
Physical activity (once a year) at time t	0.278*** (0.04)	0.272*** (0.04)	0.279*** (0.04)
Physical activity (several times a year)	0.616*** (0.03)	0.608*** (0.03)	0.613*** (0.03)
Physical activity (once a month)	0.963*** (0.03)	0.955*** (0.03)	0.960*** (0.03)
Physical activity (once a week) at time t	1.459*** (0.03)	1.454*** (0.03)	1.457*** (0.03)
Initial physical activity (once a year)	−0.047 (0.18)	−0.041 (0.18)	−0.040 (0.18)
Initial physical activity (several times a year)	−0.218** (0.09)	−0.220** (0.09)	−0.221** (0.09)
Initial physical activity (once a month)	−0.145* (0.08)	−0.147* (0.08)	−0.147* (0.08)
Initial physical activity (once a week)	0.081* (0.05)	0.083* (0.05)	0.080* (0.05)
2000	−0.012 (0.03)	−0.011 (0.03)	−0.010 (0.03)
2002	0.133*** (0.03)	0.134*** (0.03)	0.136*** (0.03)
2004	0.073*** (0.03)	0.073*** (0.03)	0.074*** (0.03)
2006	0.115*** (0.03)	0.115*** (0.03)	0.117*** (0.03)
2008	−1.993*** (0.03)	−1.995*** (0.03)	−1.995*** (0.03)
*Regional dummies*	*YES*	*YES*	*YES*
*No. individuals*	*8513*	*8513*	*8513*
*No. observations*	*27,359*	*27,359*	*27,359*

*Note*: The BHPS sample consists of head of household + Partner/Spouse every other year 1996–2008. Outcome variable is forward-looking and all covariates measured at time τ.

***p < 0.01; **p < 0.05; *p < 0.1.

**Table A3:** Coefficients of ordered probit models for physical activity measured at (τ+1) using stratified models.

	Model (I): Pooled	Model (II): Pooled	Model (III): Static RE	Model (IV): Dynamic RE
No. visits to the doctor	−0.040*** (0.002)	−0.043*** (0.003)	−0.037*** (0.004)	−0.025*** (0.003)
GPS	–	0.278** (0.159	0.482** (0.20)	0.166 (0.16)
GPS squared	–	3.098** (1.273)	3.873** (1.64)	1.893 (1.31)
Physical activity (once a year)	–	–	–	0.282*** (0.04)
Physical activity (several times a year)	–	–	–	0.617*** (0.03)
Physical activity (once a month)	–	–	–	0.965*** (0.03)
Physical activity (once a week)	–	–	–	1.465*** (0.02)
Initial physical activity (once a year)	–	–	–	−0.024 (0.18)
Initial physical activity (several times a year)	–	–	–	−0.216** (0.09)
Initial physical activity (once a month)	–	–	–	−0.145* (0.08)
Initial physical activity (once a week)	–	–	–	0.082* (0.05)
2000	−0.013 (0.03)	−0.016 (0.003)	−0.028 (0.03)	−0.009 (0.03)
2002	0.102*** (0.03)	0.100*** (0.15)	0.119*** (0.03)	0.131*** (0.03)
2004	0.100*** (0.03)	0.094*** (0.03)	0.108*** (0.03)	0.074*** (0.03)
2006	0.140*** (0.03)	0.133*** (0.03)	0.143*** (0.03)	0.113*** (0.03)
2008	−1.729*** (0.03)	−1.728*** (0.03)	−2.369*** (0.04)	−1.993*** (0.03)
*Regional dummies*	*YES*	*YES*	*YES*	*YES*
*No. individuals*	–	*–*	*8489*	*8489*
*No. observations*	*27,175*	*27,175*	*27,175*	*27,175*

*Note*: The BHPS sample consists of head of household + Partner/Spouse every other year 1996–2008. Outcome variable is forward-looking and all covariates measured at time τ. Ordered probit models with std. errors displayed in Models (I)–(III) on the sample of Model (IV) where the lag of physical activity reduces the no. of observations.

***p < 0.01; **p < 0.05; *p < 0.1.

**Table A4:** Coefficients of ordered probit models for physical activity measured at (τ+1) using past physical activity in the treatment model.

	Model (I): Pooled	Model (II): Pooled	Model (III): Static RE	Model (IV): Dynamic RE
No. visits to the doctor	−0.046*** (0.003)	−0.032*** (0.003)	−0.032*** (0.005)	−0.018*** (0.004)
GPS	–	0.296*** (0.21)	1.460*** (0.281)	0.830*** (0.22)
GPS squared	–	−1.844*** (0.39)	−2.042*** (0.512)	−1.172*** (1.40)
Physical activity (once a year)	–	–	–	0.249*** (0.04)
Physical activity (several times a year)	–	–	–	0.581*** (0.03)
Physical activity (once a month)	–	–	–	0.953*** (0.03)
Physical activity (once a week)	–	–	–	1.424*** (0.03)
Initial physical activity (once a year)	–	–	–	−0.005 (0.18)
Initial physical activity (several times a year)	–	–	–	−0.249*** (0.09)
Initial physical activity (once a month)	–	–	–	−0.139* (0.08)
Initial physical activity (once a week)	–	–	–	0.078* (0.05)
2002	0.106*** (0.03)	0.105*** (0.03)	0.138*** (0.03)	0.126 (0.03)
2004	0.117*** (0.03)	0.119*** (0.03)	0.148*** (0.03)	0.092*** (0.03)
2006	0.158*** (0.03)	0.157*** (0.03)	0.182*** (0.03)	0.130*** (0.03)
2008	−1.713*** (0.03)	−1.716*** (0.03)	−2.276*** (0.04)	−1.973*** (0.03)
*Regional dummies*	*YES*	*YES*	*YES*	*YES*
*No. individuals*	*–*	*–*	*7237*	*7237*
*No. observations*	*20,340*	*20,340*	*20,340*	*20,340*

*Note*: The BHPS sample consists of head of household + Partner/Spouse every other year 1996–2008. Outcome variable is forward-looking and all covariates measured at time τ. Ordered probit models with std. errors displayed in Models (I)–(III) on the sample of Model (IV) where the lag of physical activity reduces the no. of observations. GPS has been predicted from a treatment model that includes lagged physical activity.

***p < 0.01; **p < 0.05; *p < 0.1.

**Table A5:** Coefficients of random effects models for smoking and drinking measured at (τ+1)..

	Model (I): Cigarettes smoking at time (τ+1)	Model (II): Alcohol drinking at time (τ+1)
No. visits to the doctor	0.06** (0.03)	−0.01** (0.003)
GPS	0.40 (0.72)	0.27*** (0.06)
Cigarette smoking	0.58*** (0.02)	–
Initial cigarette smoking	0.25*** (0.02)	–
Alcohol drinking (at least once a week)	–	−1.82*** (0.05)
Alcohol drinking (at least once a month)	–	−1.40*** (0.04)
Alcohol drinking (several times a year)	–	−0.92*** (0.03)
Initial alcohol drinking (at least once a week)	–	−1.08*** (0.06)
Initial alcohol drinking (at least once a month)	–	−0.76*** (0.04)
Initial alcohol drinking (several times a year)	–	−0.36*** (0.03)
2000	0.12 (0.30)	−0.01 (0.03)
2002	0.12 (0.31)	−0.08** (0.03)
2004	−0.59* (0.31)	−0.11 (0.03)
2006	0.25 (0.32)	−0.12 (0.03)
2008	−0.67** (0.33)	−0.28 (0.03)
constant	3.17*** (0.46)	–
*Regional dummies*	*YES*	*YES*
*No. individuals*	*1165*	*7366*
*No. observations*	*3593*	*22,414*

*Note*: The BHPS sample consists of head of household + Partner/Spouse who were smokers (Model I) or drinkers. (Model II) on the first wave between 1996 and 2008 they were observed. Outcome variable is forward-looking and all covariates measured at time τ. Model I is RE OLS model and Model II is RE ordered probit model with std. errors displayed in ().

***p < 0.01; **p < 0.05; *p < 0.1.

**Table A6:** Coefficients of physical activity measured at (τ+1) with alternative econometric models.

	Model (I): Static RE model on sample with health checks	Model (II): FE model	Model (III): 2SRI model with spouse instrument	Model (IV): 2SRI model with LAD instrument
No. visits to the doctor	−0.016*** (0.005)	−0.017** (0.008)	−0.069*** (0.008)	−0.064*** (0.009)
Residuals	–	–	−0.025*** (0.004)	−0.020*** (0.005)
University degree	0.413*** (0.09)	0.605 (1.42)	0.379*** (0.06)	0.387*** (0.07)
Employed	0.052 (0.04)	−0.111*** (0.03)	−0.011 (0.04)	0.004 (0.05)
Log(Income)	0.071*** (0.03)	0.004 (0.02)	0.106*** (0.02)	0.091*** (0.02)
Age*Gender	−0.080 (0.07)	0.034 (0.04)	−0.076* (0.04)	−0.077** (0.04)
Non-single*Gender	0.066 (0.09)	−0.141*** (0.05)	–	0.090*** (0.02)
University degree*Gender	−0.07 (0.11)	−0.313* (0.19)	−0.042 (0.09)	−0.038 (0.09)
2000	−0.032 (0.05)	−0.045** (0.02)	−0.040 (0.04)	−0.041 (0.04)
2002	0.105*** (0.03)	0.138*** (0.03)	0.108*** (0.04)	0.108*** (0.05)
2004	0.058 (0.05)	0.070** (0.02)	0.105*** (0.04)	0.081*** (0.04)
2006	0.035 (0.04)	0.088*** (0.03)	0.123*** (0.04)	0.125*** (0.04)
2008	−2.103*** (0.06)	−2.346*** (0.03)	−2.396*** (0.05)	−2.381*** (0.07)
Constant	*–*	3.960*** (0.16)	–	–
*Regional dummies*	*YES*	*YES*	*YES*	*YES*
*No. individuals*	*4522*	*10,676*	*5700*	*8417*
*No. observations*	*8353*	*38,712*	*17,375*	*27,094*

*Note*: The BHPS sample consists of head of household + Partner/Spouse every other year 1996–2008. Std. errors in (), bootstrapped std. errors in Models III–IV. Std. errors in Model (IV) are clustered by the LAD. Outcome is forward-looking and all covariates measured at time τ. Models III–IV are estimated on the samples of Models in Table 5. Model I is a random effects ordered probit with all covariates used to predict the GPS, Model II is a linear model. Model III–IV are random effects ordered probit models where no. visits to the doctor is measured at the same time as physical activity but instrumented with LAD-level instrument and spouse visits instrument respectively. ***p < 0.01; **p < 0.05; *p < 0.1.

**Table A7:** Coefficients of ordered probit models for physical activity measured at (τ+1) for the population aged 60 years and over.

	Model (I): Pooled	Model (II): Pooled	Model (III): Static RE	Model (IV): Dynamic RE
No. visits to the doctor	−0.058*** (0.003)	−0.045*** (0.004)	−0.048*** (0.005)	−0.035*** (0.004)
GPS	–	2.801*** (0.41)	2.449*** (0.58)	2.320*** (0.47)
GPS squared	–	−7.195*** (1.13)	−6.632*** (1.57)	−6.165***(1.29)
Physical activity (once a week)	–	–	–	1.037*** (0.03)
Initial physical activity (once a week)	–	–	–	0.171 (0.11)
2000	−0.018 (0.04)	0.019 (0.04)	−0.052 (0.05)	0.021 (0.05)
2002	0.173*** (0.04)	0.178*** (0.04)	0.068 (0.05)	0.173*** (0.04)
2004	0.126*** (0.04)	0.129*** (0.04)	−0.088* (0.05)	0.052 (0.04)
2006	0.257*** (0.04)	0.257*** (0.04)	0.062(0.05)	0.259*** (0.04)
2008	−1.052*** (0.04)	−1.053*** (0.04)	−1.807*** (0.05)	−1.406*** (0.05)
*Regional dummies*	*YES*	*YES*	*YES*	*YES*
*No. individuals*	*–*	*–*	*4678*	*4678*
*No. observations*	*14,753*	*14,753*	*14,753*	*14,753*

*Note*: The BHPS sample consists of head of household + Partner/Spouse aged 60+ every other year 1996–2008. Outcome variable is forward-looking and all covariates measured at time τ. Ordered probit models with std. errors displayed in Models (I)–(III) on the sample of Model (IV) where the lag of physical activity reduces the no. of observations.

***p < 0.01; **p < 0.05; *p < 0.1.

**Table 1:** Summary Statistics.

	No. obs.	Mean	Min.	Max.	S.D.
No. visits to the doctor(τ+1):					
None	11,265	0.27			
One to two	15,490	0.37			
Three to five	7729	0.19			
Six to ten	3636	0.09			
More than ten	3213	0.08			

Physical activity(τ+1):					
Never/almost never	10,870	0.26			
Once a year or less	1767	0.04			
Several times a year	3930	0.09			
At least once a month	5045	0.12			
At least once a week	19,721	0.48			

*Pre-treatment variables:*(τ)					
Age 45+	38,714	0.46	0	1	
White	38,714	0.99	0	1	
Black	38,714	0.003	0	1	
Asian	38,714	0.005	0	1	
Other ethnicity	38,714	0.002	0	1	
Male	38,714	0.55	0	1	
Married/cohabiting	38,714	0.81	0	1	
University degree	38,714	0.16	0	1	
High school diploma	38,714	0.51	0	1	
Higher National Diploma	38,714	0.08	0	1	
No qualification	38,714	0.25	0	1	
No. children 0–2	38,714	0.08	0	3	0.28
Employed	38,714	0.78	0	1	
Log(Income)	38,714	6.52	0	10.26	0.75
No. rooms in the house	38,714	5	1	45	1.7
Initial no. health conditions (0–2)	38,714	0.93	0	1	
Initial no. health conditions (3)	38,714	0.04	0	1	
Initial no. health conditions (4)	38,714	0.02	0	1	
Initial no. health conditions (5)	38,714	0.003	0	1	
Initial no. health conditions (6+)	38,714	0.002	0	1	
Initial musculoskeletal	38,714	0.16	0	1	
Initial cardiovascular disease	38,714	0.06	0	1	
Initial diabetes	38,714	0.07	0	1	
Initial skin, head and sight problems	38,714	0.17	0	1	
Initial respiratory problems	38,714	0.09	0	1	
Initial stomach problems	38,714	0.05	0	1	
Initial depression	38,714	0.14	0	1	
Initial other health problem	38,714	0.05	0	1	
London	38,714	0.19	0	1	
South East	38,714	0.13	0	1	
South West	38,714	0.06	0	1	
East Anglia	38,714	0.03	0	1	
East Midlands	38,714	0.06	0	1	
West Midlands	38,714	0.05	0	1	
North West	38,714	0.07	0	1	
Yorkshire	38,714	0.06	0	1	
Rest of the North	38,714	0.04	0	1	
Wales	38,714	0.14	0	1	
Scotland	38,714	0.17	0	1	

*Note*: The BHPS sample consists of head of household + Partner/Spouse every other year 1996–2008. All sample sizes refer to the sample of treatment, outcome and covariates. Outcome variable is forward-looking. Number and type of conditions are measured at initial time τ. Interactions between health conditions, age and education not displayed.

**Table 2:** Coefficients of random effects grouped Poisson model for visits to the doctor measured at (τ+1)..

	Visits to the doctor (τ+1)	
	Coeff.	Std. Err.
No. visits to the doctor (1–5) at time τ	0.46***	0.01
No. visits to the doctor (6+) at time τ	0.85***	0.01
Age 45+	0.06**	0.02
Male	0.22***	0.04
White	−0.10	0.15
Black	−0.17	0.18
Asian	0.05	0.17
Non-single	−0.05*	0.03
University degree	−0.49	0.78
High school diploma	0.004	0.02
No. children 0–2	−0.07***	0.01
Employed	−0.01	0.01
Log(Income)	0.04***	0.01
No. rooms in the house	−0.01*	0.004
No. initial visits to the doctor (1–5)	0.28***	0.02
No. initial visits to the doctor (6+)	0.53***	0.03
No. initial health conditions (3)	−0.01	0.04
No. initial health conditions (4)	−0.10*	0.06
No. initial health conditions (5)	−0.15	0.10
No. initial health conditions (6+)	−0.28**	0.13
Initial musculoskeletal condition	0.21***	0.02
Initial CVD condition	−0.20**	0.10
Initial diabetes condition	0.43***	0.09
Initial skin, head and sight problems	0.06***	0.02
Initial respiratory problems	0.14***	0.03
Initial stomach problems	0.22***	0.04
Initial depression condition	0.15***	0.03
South East	−0.05*	0.02
South West	−0.04	0.03
East Anglia	0.004	0.05
East Midlands	0.03	0.03
West Midlands	0.03	0.03
North West	0.02	0.03
Yorkshire	0.03	0.03
Rest of the North	0.04	0.04
Wales	−0.02	0.02
Scotland	0.002	0.02
1998	−0.01	0.01
2000	0.001	0.01
2002	−0.01	0.01
2004	−0.09***	0.01
2006	−0.06***	0.01
2008	−0.04***	0.02
*Includes means of time-varying covariates*	*YES*	
Constant	0.57***	0.17

*No. individuals*	*10,686*	
*No. observations*	*38,714*	

*Note*: The BHPS sample consists of head of household + Partner/Spouse every other year 1996–2008. Outcome variable is forward-looking and all covariates measured at time τ. Number and type of conditions are measured at initial time τ. Interactions between health conditions, age and education not displayed.

***p < 0.01; **p < 0.05; *p < 0.1.

**Table 3:** Balancing tests not adjusted for the GPS.

	Cut 1: [0,0]	Cut 2: [1,5]	Cut 3: [6+]
No. visits to the doctor (1–5)	−19.4***	31.3***	−18***
No. visits to the doctor (6+)	−36.8***	−17.2***	47***
Age 45+	−7***	−1.4	10.3***
Male	−32.8***	10.2***	27***
White	2.6***	1.0	−3.7***
Black	−0.6	0.3	0.3
Asian	−2.3	−1.5	3.7***
Non-single	7.5***	4.2***	−13.3***
University degree	5.9***	5.1***	−16.5***
High school	1.5	4.0***	−7.2***
No. children 0–2	2.3	−0.5	−2.2
Employed	25.4***	14***	−40.2***
Log(Income)	7.4***	8.1***	−19.8***
No. rooms in the house	8.7***	5.6***	−18.1***
No. initial visits to the doctor (1–5)	−14.4***	27.6***	−19.2***
No. initial visits to the doctor (6+)	−39.6***	−14.1***	46.3***
No. health conditions (3)	−20***	−4.9***	18.1***
No. health conditions (4)	−16.2***	−7.7***	15.2***
No. health conditions (5)	−10***	−5.6***	8.6***
No. health conditions (6+)	−7.8***	−6.1***	7.6***
Initial musculoskeletal condition	−22***	−5.4***	26***
Initial CVD condition	−20.3***	−2.6***	17.7***
Initial diabetes condition	−21.4***	−3.5***	19.6***
Initial skin, head and sight problems	−13.7***	2.2	11.6***
Initial respiratory problems	−15.9***	−1.5	15.8***
Initial stomach problems	−16.8***	−3.7***	16.4***
Initial depression condition	−25.1***	−4.6***	26.5***
South East	2.4	3.6***	−8.5***
South West	2.5	1.2	−5.2***
East Anglia	1.9	0.5	−3.2***
East Midlands	0.5	−0.3	−0.2
West Midlands	−0.2	−0.9	1.4
North West	−0.2	0.2	0
Yorkshire	−1.8	0.9	0.9
Rest of the North	0.5	−3.6***	3.9***
Wales	2.4	−4.1***	2.5
Scotland	−1.5	−1.2	3.2***
1998	−2.6***	2.6***	−0.4
2000	−0.8	−1.1	2.4
2002	−1.9	−1.1	3.6***
2004	3.1***	−0.4	−3.3***
2006	2.9***	−1.5	−1.5
2008	−0.9	1.9	−1.5

*Note*: The BHPS sample consists of head of household + Partner/Spouse every other year 1996–2008. Number and type of conditions are measured at initial time τ. t-stats displayed. Interactions between health conditions, age and education not displayed.

***p < 0.01.

**Table 4:** Balancing tests adjusted for the GPS.

	Cut 1: [0,0]	Cut 2: [1,5]	Cut 3: [6+]
No. visits to the doctor (1–5)	−0.8	2.5	−0.3
No. visits to the doctor (6+)	−2.1	−1.5	2.4
Age 45+	−0.1	−0.3	0.3
Male	−0.8	0.4	1.8
White	0.0	0.0	−0.1
Black	0.0	0.0	0.0
Asian	0.0	0.0	0.0
Non-single	0.3	0.3	−0.5
University degree	−0.2	0.6	−0.6
High school	0.2	0.3	−0.2
No. children 0–2	−0.1	0.0	−0.1
Employed	0.9	1.1	−1.5
Log(Income)	0.1	0.8	−1.0
No. rooms in the house	0.2	1.1	−1.7
No. initial visits to the doctor (1–5)	0.6	1.7	−0.1
No. initial visits to the doctor (6+)	−2.1	−1.1	1.6
No. health conditions (3)	−0.8	−0.2	0.6
No. health conditions (4)	−0.6	−0.4	0.4
No. health conditions (5)	−0.3	−0.2	0.1
No. health conditions (6+)	−0.2	−0.2	0.1
Initial musculoskeletal condition	−0.9	−0.4	1.0
Initial CVD condition	−0.8	−0.1	0.6
Initial diabetes condition	−0.9	−0.2	0.7
Initial skin, head and sight problems	−0.5	0.1	0.5
Initial respiratory problems	−0.7	−0.1	0.5
Initial stomach problems	−0.6	−0.2	0.5
Initial depression condition	−1.1	−0.3	1.2
South East	0.0	0.3	−0.3
South West	0.0	0.1	−0.2
East Anglia	0.0	0.0	0.0
East Midlands	0.0	0.0	0.0
West Midlands	0.0	−0.1	0.0
North West	0.0	0.0	0.0
Yorkshire	0.1	0.0	0.1
Rest of the North	0.1	−0.2	0.2
Wales	0.2	−0.3	0.1
Scotland	0.0	−0.1	0.0
1998	−0.1	0.2	0.0
2000	0.0	−0.1	0.1
2002	−0.1	−0.1	0.1
2004	0.2	0.0	−0.3
2006	0.2	−0.1	0.0
2008	−0.1	0.2	0.1

*Note*: The BHPS sample consists of head of household + Partner/Spouse every other year 1996–2008. Number and type of conditions are measured at initial time τ. t-stats displayed. Interactions between health conditions, age and education not displayed.

***p < 0.01*.*

**Table 5:** Coefficients of ordered probit models for physical activity measured at (τ+1)..

	Model (I): Pooled	Model (II): Pooled	Model (III): Static RE	Model (IV): Dynamic RE
No. visits to the doctor	−0.044*** (0.002)	−0.034*** (0.003)	−0.028*** (0.004)	−0.019*** (0.003)
GPS	–	1.166*** (0.19)	1.404*** (0.25)	0.776*** (0.20)
GPS squared	–	−1.881*** (0.35)	−2.152*** (0.48)	−1.239*** (0.37)
Physical activity (once a year) at time t	–	–	–	0.279*** (0.04)
Physical activity (several times a year)	–	–	–	0.612*** (0.03)
Physical activity (once a month)	–	–	–	0.960*** (0.03)
Physical activity (once a week) at time t	–	–	–	1.456*** (0.03)
Initial physical activity (once a year)	–	–	–	−0.039 (0.18)
Initial physical activity (several times a year)	–	–	–	−0.221** (0.09)
Initial physical activity (once a month)	–	–	–	−0.147* (0.08)
Initial physical activity (once a week)	–	–	–	0.080* (0.05)
2000	−0.016 (0.03)	−0.017 (0.03)	−0.028 (0.03)	−0.010 (0.03)
2002	0.104*** (0.03)	0.106*** (0.03)	0.127*** (0.03)	0.136*** (0.03)
2004	0.095*** (0.03)	0.095*** (0.03)	0.108*** (0.03)	0.074*** (0.03)
2006	0.137*** (0.03)	0.136*** (0.03)	0.146*** (0.03)	0.117*** (0.03)
2008	−1.726*** (0.03)	−1.728*** (0.03)	−2.366*** (0.04)	−1.995*** (0.03)
*Regional dummies*	*YES*	*YES*	*YES*	*YES*
*No. individuals*	*–*	*–*	*8513*	*8513*
*No. observations*	*27,359*	*27,359*	*27,359*	*27,359*

*Note*: The BHPS sample consists of head of household + Partner/Spouse every other year 1996–2008. Outcome variable is forward-looking and all covariates measured at time τ. Ordered probit models with std. errors displayed in Models (I)–(III) on the sample of Model (IV) where the lag of physical activity reduces the no. of observations.

***p < 0.01; **p < 0.05; *p < 0.1.

**Table 6:** Marginal effects of no. visits to the doctor from random effects ordered probit models.

	*PA_1_*	*PA_2_*	*PA_3_*	*PA_4_*	*PA_5_*
	Never	Once a year	Several times a year	Once a month	Once a week
No. visits to the doctor (Model III)	0.0050*** (0.0007)	0.0009*** (0.0001)	0.0019*** (0.0003)	0.0013*** (0.0002)	−0.0091*** (0.0013)
*–*					
No. visits to the doctor (Model IV)	0.0041*** (0.0007)	0.0004*** (0.0001)	0.0006*** (0.0001)	0.0004*** (0.0001)	−0.0054*** (0.0009)
*–*					
*No. obs.*	*27,359*				

*Note*: The BHPS sample consists of head of household + Partner/Spouse every other year 1996–2008. ***p < 0.01.

## Footnotes

1 Brown et al. (2014) used a zero-inflated model. Teckle and Sutton (2008) compare Poisson and negative binomial models and show the results are not very different. As we are not actually interested in the treatment model, but we only use it to predict the propensity score, we use a simple Poisson model.

2 See Contayannis et al. (2004) for an empirical application to self-assessed health.

3 Because individuals move between LADs, panels are not nested within clusters. Therefore we clustered standard errors by the LAD individuals were first observed to live in.

4 We do not report the results of Eq. (1) because they are the same as the treatment model reported in Table 2.

5 All interactions have been omitted from the table in the interest of space.
